# Using a meta-narrative literature review and focus groups with key stakeholders to identify perceived challenges and solutions for generating robust evidence on the effectiveness of treatments for rare diseases

**DOI:** 10.1186/s13023-018-0851-1

**Published:** 2018-06-28

**Authors:** Kylie Tingley, Doug Coyle, Ian D. Graham, Lindsey Sikora, Pranesh Chakraborty, Kumanan Wilson, John J. Mitchell, Sylvia Stockler-Ipsiroglu, Beth K. Potter, in collaboration with the Canadian Inherited Metabolic Diseases Research Network

**Affiliations:** 1 School of Epidemiology and Public Health, University of Ottawa, 600 Peter Morand Crescent, Ottawa, ON K1G 5Z3 Canada; 2 Ottawa Hospital Research Institute, Ottawa, ON Canada; 3 Health Sciences Library, University of Ottawa, Ottawa, ON Canada; 4 Metabolics and Newborn Screening, Department of Pediatrics, Children’s Hospital of Eastern Ontario, Ottawa, ON Canada; 5 Department of Pediatrics, University of Ottawa, Ottawa, ON Canada; 6 Newborn Screening Ontario, Ottawa, ON Canada; 7 Department of Pediatrics and Department of Medical Genetics, McGill University Health Centre, Montreal, QC, Canada; 8 Division of Biochemical Diseases, BC Children’s Hospital, Vancouver, BC Canada; 9 Department of Pediatrics, University of British Columbia, Vancouver, BC Canada

**Keywords:** Rare diseases, Evidence generation, Comparative effectiveness, Patient-oriented outcomes, Evidence synthesis, Research methods

## Abstract

**Introduction:**

For many rare diseases, strong analytic study designs for evaluating the efficacy and effectiveness of interventions are challenging to implement because of small, geographically dispersed patient populations and underlying clinical heterogeneity. The objective of this study was to integrate perspectives from published literature and key rare disease stakeholders to better understand the perceived challenges and proposed methodological approaches to research on clinical interventions for rare diseases.

**Methods:**

We used a meta-narrative literature review and focus group interviews with key rare disease stakeholders to better understand the perceived challenges in generating and synthesizing treatment effectiveness evidence, and to describe various research methods for mitigating these identified challenges. Data from both components of this study were synthesized narratively according to research paradigms that emerged from our data.

**Results:**

Results from our meta-narrative literature review and focus group interviews revealed three fundamental challenges in generating robust treatment effectiveness evidence for rare diseases: i) limitations in recruiting a sufficient sample size to achieve planned statistical power; ii) inability to account for clinical heterogeneity and assess treatment effects across a clinical spectrum; and iii) reliance on short-term, surrogate outcomes whose clinical relevance is often unclear. We mapped these challenges and associated solutions to three interrelated research paradigms: i) explanatory evidence generation; ii) comparative effectiveness/pragmatic evidence generation; and iii) patient-oriented evidence generation. Within each research paradigm, numerous criticisms and potential solutions have been described with respect to overcoming these challenges from a research study design perspective.

**Conclusions:**

Over time, discussions about clinical research for interventions for rare diseases have moved beyond methodological approaches to overcome challenges related to explanatory evidence generation, with increased recognition of the importance of pragmatic and patient-oriented evidence. Future directions for our work include developing a framework to expand current evidence synthesis practices to take into consideration many of the concepts discussed in this paper.

**Electronic supplementary material:**

The online version of this article (10.1186/s13023-018-0851-1) contains supplementary material, which is available to authorized users.

## Background

For many rare diseases, strong analytic study designs for evaluating the *efficacy* (does intervention X work under *ideal conditions*?) and *effectiveness* (does intervention X work in *real-world practice*?) [1] of interventions are challenging to implement because of small, geographically dispersed patient populations and characteristically high clinical heterogeneity [2]. A poor understanding of natural history for many rare diseases, scarcity of validated measures of disease progression, and various financial constraints (e.g., limited availability of research funding, high costs of trials for rare diseases) also add to the complexity of evaluating treatments for rare diseases [2–4]. As a result of these limitations, it is often not feasible to conduct conventional randomized controlled trials (RCTs), the gold standard for determining treatment efficacy [5]. Thus, rare disease researchers must often rely on other study designs that are more prone to bias when evaluating interventions, such as open label or uncontrolled trials, observational studies, and case reports. [6, 7]

The evidence that exists for clinical interventions for rare diseases therefore typically falls in the bottom half of the traditional evidence hierarchy [7, 8] and is methodologically flawed [6, 9]. For example, a recent systematic review of available evidence for 11 orphan medicines found that case studies represented the largest proportion (140/338; 41%) of study designs used to determine clinical effectiveness, while only 7% (14/338) of studies were double-blind, placebo-controlled RCTs [6]. Studies that have reviewed the evidence for clinical interventions for rare diseases that is submitted to regulatory and health technology assessment agencies in support of marketing authorization and reimbursement approval have also found limited RCT evidence for some rare diseases, particularly those considered ‘ultra-rare’ [10–13]. Newer processes for both regulatory approval and reimbursement approval may be shifting the standards in terms of evidence requirements in this rapidly evolving area [14–16]. A recent analysis of ClinicalTrials.gov comparing characteristics of completed or on-going trials for rare and non-rare disease treatments demonstrated that trials for rare disease therapies are likely to enroll fewer participants, be single arm, non-randomized, and open label [17], all of which can compromise the internal validity of a study.

The lack of high quality evidence and the typically high cost of clinical interventions for rare diseases commonly result in debates about the efficacy and effectiveness of these interventions among stakeholders [18, 19]. Disagreements about the evidence arise from differing views about the methodological rigour of the study design; what constitutes a meaningful outcome; and the minimal clinically important difference for a relevant outcome [20]. Disputes among stakeholders are further fueled by differing values and the institutional/political landscape surrounding decision-making processes about interventions for rare diseases [20, 21]. As a result, health policy recommendations, such as those concerning reimbursement for some clinical interventions for rare diseases, are variable across jurisdictions [22, 23].

The objective of this study was to integrate perspectives from published literature and key rare disease stakeholders to better understand the challenges and approaches to research for clinical interventions for rare diseases. More specifically, we sought to:

1. identify perceived challenges in generating robust evidence for establishing treatment efficacy and effectiveness in the context of rare diseases; and
2. describe various clinical evaluative research methods that have been suggested for mitigating the identified challenges in generating robust evidence, focusing on the perceived strengths and limitations specific to each.

## Methods

### Meta-narrative literature review

#### Meta-narrative approach

An initial scoping of the literature regarding our research topic revealed diverse perspectives on generating evidence for efficacy and effectiveness of treatments for rare diseases. We therefore chose to use an adaptation of the meta-narrative approach developed by Greenhalgh and colleagues specifically for systematically reviewing the literature on complex topics that have been conceptualized and studied differently among researchers [24]. Meta-narrative reviews encompass six main principles: (1) *pragmatism*, the included information should be driven by usefulness to the intended audience; (2) *pluralism*, the topic should be considered from multiple perspectives; (3) *historicity*, the included information should be presented according to its development over time; (4) *contestation*, any conflicting information should be used to generate higher-order insights; (5) *reflexivity*, there should be continual reflection on the review findings; and (6) *peer review*, the review findings should be presented to an external audience for feedback [24, 25]. Below we describe the methods for each phase of our review separately and sequentially, while recognizing that the phases overlap with each other [24].

#### Planning and search phases

Our interdisciplinary study team has expertise in epidemiology, health services research, health economics, and information science. We held a series of meetings to discuss the emerging findings from the literature and provide direction as the project progressed. We also agreed that the outputs from this review would include a summary of current knowledge across research paradigms on the topic of establishing efficacy or effectiveness for clinical interventions for rare diseases, and a framework to guide future evidence syntheses in this field (*currently under development*).

We used an initial exploratory search (snowball sampling and citation-searching) to identify important sources of information relevant to our study objectives, and in turn, developed a formal search strategy comprised of Medical Subject Heading (MeSH) terms and keywords. Our search strategy was developed iteratively (by LS and KT) and was not meant to be exhaustive, but was designed to identify *key* sources of scholarly information. Three electronic databases were searched: MEDLINE (Ovid MEDLINE (R) In-Process & Other Non-Indexed Citations and Ovid MEDLINE (R) 1946 to June 21, 2017), EMBASE (Embase Classic + Embase 1947 to June 21, 2017), and PubMed. Search strategies for each database can be found in Additional file 1. We also scanned reference lists from included studies for any additional citations.

All citations returned from the searches were reviewed using a two-stage approach. During the first stage, one member of the study team (KT) scanned titles and abstracts of all citations to identify potentially relevant records. For the second stage, full-text articles were retrieved for all citations identified during stage one, and one member of the study team (KT) reviewed the articles to determine final inclusion/exclusion. Given that the purpose of the search and screening phases was to identify *key* sources of information rather than to be exhaustive, and that the meta-narrative approach is reflexive by design, having only one member of the team screen citations and papers for eligibility was deemed appropriate. To help mitigate bias, we established the following inclusion criteria: (i) relevant to rare diseases or orphan medicines; and (ii) describes methods used to overcome challenges for establishing efficacy or effectiveness of clinical interventions for rare diseases. We did not limit inclusion to primary studies (i.e., review articles were included), but did exclude letters to the editor, conference abstracts, and commentaries. We also did not limit according to specific diseases or disease groups. Finally, given language constraints within the team, we excluded all articles not written in English.

#### Mapping, appraisal and synthesis phases

A fundamental aspect of the meta-narrative approach is constructing a story about how research on a given topic has unfolded over time [24, 25]. To this end, we extracted information from each report to identify key people, events, research questions, conceptual and theoretical issues, research findings, and areas of debate or disagreement. Data extracted from each study included (if applicable): bibliographic characteristics (publication date, author(s), geographical location), sponsorship/declared conflicts of interest, and report characteristics (type of study, disease(s) of interest, study objectives, main findings/conclusions, etc.). Additionally, we used the following guiding questions to extract further information to describe the different perspectives:

1. What study designs have been described for studying the efficacy or effectiveness of treatments for rare diseases?
2. What strengths, weaknesses, and risks of bias are reported as important for each study design?
3. What are the described tradeoffs in risk of bias among the study designs?
4. Is the choice of outcome(s) reported as an influence on the quality of evidence?

Data were extracted from each report by a single reviewer (KT) and findings were reviewed and discussed at team meetings. Bibliographic and report characteristics were synthesized descriptively, and all other study findings were synthesized narratively.

### Focus group interviews with stakeholders

#### Design, sampling, recruitment, and participants

In parallel with the meta-narrative review, we conducted focus group interviews with three stakeholder groups to better understand their perspectives on generating evidence for clinical interventions for rare diseases. We recruited a convenience sample from three groups who could speak knowledgeably (based on formal knowledge or experience) about evidence for interventions for rare diseases, including: physicians, policy advisors, and rare disease patients or caregivers. More specifically, we chose to include rare disease patients and caregivers as stakeholders because they are directly impacted by clinical research and could provide unique perspectives based on their lived experiences, especially in regards to outcomes that reflect quality of life and in considering how the selection of outcomes affects the relevance of the evidence produced. To facilitate the focus group discussions with physicians and patients/caregivers, we chose rare inherited metabolic diseases as a case study. For the patients/caregivers, we further narrowed the selection to mucopolysaccharidoses (MPS), a group of rare metabolic conditions, because this group of diseases typifies the characteristics of many rare diseases that present challenges for conducting strong analytic studies, including low prevalence (i.e., very small population), significant clinical heterogeneity, and, for some MPS types, the existence of expensive orphan drug treatments that require evaluation. In addition, this restriction supported an in-person discussion with patients/caregivers as we could meet with that group as part of an annual event (described below). We sought between five and eight participants per focus group, based on standard focus group interview methodology [26]. Individuals were eligible to participate if they had experience with the care of those diagnosed with a rare inherited metabolic disease (metabolic physicians), if they had experience in evidence review activities that result in recommendations being made about the development, use, and/or reimbursement of interventions for rare diseases (policy advisors) or if they were adults diagnosed with MPS or a related disease, or were the caregiver (i.e., parent/guardian) of someone diagnosed with MPS or a related disease.

Recruitment invitations were distributed by email to physician members of the Garrod Association (a professional association whose members are involved in caring for patients with inherited metabolic diseases), to policy advisors by a member of their professional network (using publicly available contact information), and to patients/caregivers attending the Canadian MPS Society’s 2017 Annual Family Meeting. Individuals interested in participating were instructed to contact a member of the research team (KT), and eligible respondents were asked to provide signed, informed consent to participate in the study. Focus group interviews were conducted by telephone with the physicians and policy advisors, and in-person with the patients/caregivers in conjunction with the Canadian MPS Society’s 2017 Annual Family Meeting held in Montreal, QC, Canada. The study protocol was approved by the Ottawa Health Science Network Research Ethics Board and the Children’s Hospital of Eastern Ontario Research Ethics Board (physicians and policy advisors), and the University of Ottawa Health Sciences and Sciences Research Ethics Board (patients/caregivers).

#### Data collection

Focus group interviews were conducted by a single member of the study team (KT) using a semi-structured interview guide and were attended by a second member of the team as an observer (BKP or JJM). The interview guide was tailored to the specific stakeholder group. The interview guide addressed general perspectives on the challenges of rare disease research, and more specific topics including generation and synthesis of evidence to establish treatment efficacy or effectiveness, and outcomes used in clinical evaluative studies. All interviews were audio-recorded with participants’ consent and subsequently transcribed.

#### Data analysis

Each focus group transcript was analyzed using a qualitative descriptive approach that is aimed at *“obtaining straight and largely unadorned (i.e., minimally theorized or otherwise transformed or spun) answers to questions of special relevance to practitioners and policy makers”* [27]. Four members of the study team (KT, BP, DC, IG) met to identify the key concepts and themes that were present in the focus group data. These concepts/themes were organized into a coding system that was applied by one study team member (KT) using NVivo 10 Software (QSR International Pty Ltd.) and reviewed by a second member (BP) for credibility and trustworthiness [28].

## Results

### Search & screening results

**Fig. 1:**
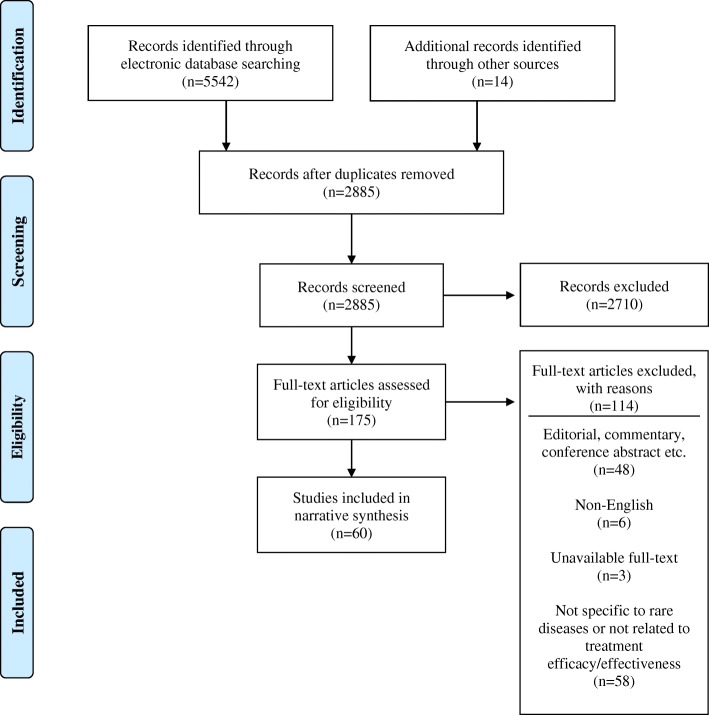
PRISMA flow diagram outlining results from search and screening process. (Adapted from: Liberati et al. 2009) [29]

Electronic database searches returned 2871 records after removal of duplicates, of which 161 records were identified as potentially relevant based on the title and abstract scan. An additional 14 titles were identified as potentially relevant from scanning reference lists of included studies. Full text articles were successfully obtained for 172/175 records. Of the 172 full-text articles reviewed, 60 articles were included in this review (Fig. 1 [29]).

### Descriptive study characteristics

**Fig. 2:**
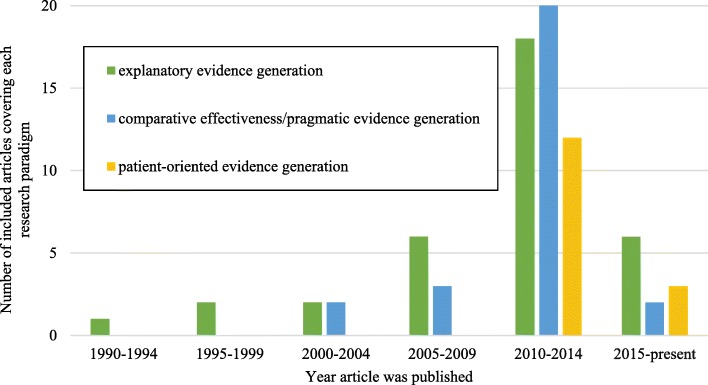
Research paradigms discussed by year of publication (note: research paradigms are not mutually exclusive)

Of the 60 articles we reviewed, 57 (95%) were published after 2000 (Table 1; Fig. 2). Based on the location of the corresponding author address, 27/60 articles (45%) were written by authors from the United States, 8 (13%) from authors in Canada, 5 (8%) from authors in the United Kingdom, and the remaining articles were written by authors across Europe and Australia (Table 1). Sixteen (27%) articles explicitly reported that their study was sponsored by industry or had some affiliation with industry, while conflicts of interest/study sponsorship were not explicitly reported for a further 14 (23%). Over half of the included studies (33/60; 55%) reported on rare diseases in general, while the remaining articles focused on a specific disease or group of diseases. A majority of the articles included were review articles of research methods used to evaluate efficacy or effectiveness of interventions for rare diseases (39/60; 65%); however, 28% (17/60) described the application of a specific research method in the rare disease context (Table 1). While most of the articles reviewing methods were focused on rare diseases generally (26/39, 67%), many of the applied studies were specific to single diseases (13/17, 76%). A list of the included articles can be found in Additional file 2.

**Table 1:** Descriptive characteristics of included studies (*n* = 60)

Study characteristic	Number of studies (%)
Year of publication	
1990–1994	1 (2)
1995–1999	2 (3)
2000–2004	3 (5)
2005–2009	7 (12)
2010–2014	39 (65)
2015-present (June 21, 2017)	8 (13)
Country (corresponding author address)	
United States	27 (45)
United Kingdom	5 (8)
Canada	8 (13)
France	4 (7)
The Netherlands	6 (10)
Germany	3 (5)
Italy	4 (7)
Belgium	1 (2)
Ireland	1 (2)
Australia	1 (2)
Sponsorship	
Industry affiliations	16 (27)
None (no conflicts of interest declared)	24 (40)
Other (e.g., government funding)	6 (10)
Not explicitly reported	14 (23)
Disease/disease group of focus	
Rare diseases in general	33 (55)
Disease groups:	
Inherited metabolic diseases^a^	2 (3)
Lysosomal storage disorders	3 (5)
Pediatric rheumatic diseases	1 (2)
Rare lung diseases	2 (3)
Rare neonatal diseases	1 (2)
Rare neurodegenerative diseases	1 (2)
Rare renal diseases	1 (2)
Individual disease(s):	
Alpha1-antitrypsin deficiency & pulmonary alveolar proteinosis	1 (2)
Batten Disease	1 (2)
Childhood polyarteritis nodosa	2 (3)
Duchenne muscular dystrophy	1 (2)
Familial hypercholesterolemia	1 (2)
Familial Mediterranean fever	1 (2)
Gaucher disease	1 (2)
Hemophilia A	1 (2)
Late-onset Pompe disease	1 (2)
Non-dystrophic mytonia	1 (2)
Pediatric multiple sclerosis & Creutzfeldt-Jakob disease	1 (2)
Primary sclerosing cholangitis	1 (2)
Scleroderma	2 (3)
Vasculitis (rare form)	1 (2)
Types of studies	
Review article of multiple research methods	28 (47)
Review article of a single research method	11 (18)
Application/case example of research method	17 (28)
Key article that operationalized steps for the research method	3 (5)
Other	1 (2)
Research paradigm discussed^a^	
Explanatory evidence generation	35 (58)
Comparative effectiveness/pragmatic evidence generation	30 (50)
Patient-oriented evidence generation	15 (25)

^a^not mutually exclusive as some studies discussed more than one research paradigm

### Description of focus group participants

We held three focus group interviews with 13 participants in total (physicians *n* = 6; policy advisors *n* = 3; patients/caregivers *n* = 4). Across the three groups there were 9 women and 4 men. Participants were from 5 provinces in Canada: British Columbia, Alberta, Ontario, Quebec, and Newfoundland and Labrador.

### Research paradigms in establishing efficacy or effectiveness of clinical interventions for rare diseases

Three overlapping research paradigms through which stakeholders view the challenges and prioritize potential solutions for establishing efficacy or effectiveness of clinical interventions for rare diseases emerged from our data: (1) explanatory evidence generation, (2) comparative effectiveness/pragmatic evidence generation, and (3) patient-oriented evidence generation. The findings from our literature review and focus group interviews are discussed according to each of these paradigms. While each research paradigm is discussed separately, they are not mutually exclusive. A summary of perspectives across the three research paradigms is provided in Table 2.

**Table 2:** Summary of findings according to each research paradigm

Key research paradigms	Main perspectives from the literature	Main perspectives from focus group participants
Explanatory evidence generation	- internal validity for conventional RCTs in rare diseases is threatened because of the small and often heterogeneous patient population - with small numbers of participants, there is potential for unbalanced confounders across groups despite randomization, and poor statistical power to detect treatment effects- modifications to conventional RCTs have been proposed to help maximize internal validity (e.g., adaptive trials, application of Bayesian statistics) - lack of patient/family/clinician acceptance to the possibility of being randomized to a placebo group, study designs that make participation more appealing by maximizing time spent on- or guaranteeing provision of- the new treatment have been suggested (e.g., crossover trials, N-of-1 studies)	- challenging to evaluate evidence with small sample sizes and short duration of follow-up *“I do find it quite difficult when the clinical trials are very short, very small numbers, and the endpoints are something like the six minute walk test in regards to really being confident that that is going to be an effective treatment for the patients that I’m seeing.” – Physician 4* *“Well the ex-[profession] in me looks at things like, you know, the size of the study, well MPS is [laughter around the table], okay that’s not going to happen. You know, so you have to, it’s hard when you’re looking at MPS because the things that you would normally look for in a good study aren’t going to be there because of the size of the sample…” – Patient/caregiver 3*
Comparative effectiveness/pragmatic evidence generation	- external validity is threatened in rare disease research by the desire to enroll homogenous groups of participants for explanatory trials (patient population is often inherently heterogeneous) - there are also substantial differences between trial protocols and real-world clinical practice, reducing the external validity of some studies (i.e., need to account for co-interventions, less frequent follow-up visits, etc.) - several study designs have been suggested that may compromise internal validity to some extent in order to address real-world effectiveness (e.g., pragmatic trials, registry studies, hybrid study designs)	- need to address heterogeneity in treatment effect across clinical spectrum *“…the way that the trials are designed, very sub, select populations with the actual disease of concern, which is already a narrow disease as it is. It makes it very difficult for us to know where and when these therapies are going to work. And so, when we’re talking about rare diseases, it really has to be linked to not just research, but effectiveness research about natural history and epidemiology. And given the wide degree of heterogeneity with the diseases that we’re dealing with, we’re going into this with a huge degree of uncertainty about whether or not there really is any evidence to support that these therapies are going to work.” – Policy advisor 2*
Patient-oriented evidence generation	- explanatory study designs tend to rely on short-term, surrogate outcomes that are not necessarily clinically meaningful or relevant to patients and/or families - important to engage with patients and/or families throughout the research process to define important outcomes and determine minimal clinically important difference- there has been a shift/push to improve use of more patient-oriented outcomes in clinical evaluative research for rare diseases (e.g., quality of life measures, activities of daily living)- more difficult to use patient-oriented outcomes because tools have not been standardized in rare disease populations	- very important to measure outcomes that are clinically relevant *“For me I think one of the big issues is the outcome measures that we’re trying to document. For instance, with the lysosomal storage diseases, what is the relevance of a 6 min walk test? What is the clinical relevance of this type of test?” – Physician 5* *“…because yes, scientific research is important too, but it’s this push-pull dichotomy between the happiness, the living life, just the simple moments, you know, going outside, sitting in the sun, that type of, going down to the beach, those things need to be equally measured…” – Patient/caregiver 4*

#### Explanatory evidence generation

Much of the discussion in the literature and amongst focus group participants concerning evaluative clinical research in rare diseases centered on problems associated with the inherent small numbers of patients available for study and adequate recruitment for conventional RCTs, long considered the gold standard explanatory design with a low risk of bias [5].

*“I do find it quite difficult when the clinical trials are very short, very small numbers, and the endpoints are something like the six minute walk test in regards to really being confident that that is going to be an effective treatment for the patients that I’m seeing.”* – Physician 4

*“For common diseases, there’s no reason for not doing a randomized controlled trial. I mean, that’s one of the big points in the paper that we published a few years ago was that in order to be called rare, you should not have enough patients to confidently determine whether a treatment is efficacious or not.”* – Policy Advisor 1

*“Well the ex-[profession] in me looks at things like, you know, the size of the study, well MPS is [laughter around the table], okay that’s not going to happen. You know, so you have to, it’s hard when you’re looking at MPS because the things that you would normally look for in a good study aren’t going to be there because of the size of the sample…”* – Patient/caregiver 3

This paradigm was discussed by more than half (35/60, 58%) of the studies we reviewed, and was also the first that emerged in the literature in 1992 (Fig. 2). Most of the reports that discussed this paradigm were methodological review articles that focused on rare diseases in general or a group of rare diseases (24/35, 68%), rather than a single specific rare disease. The first author to highlight the challenges associated with fewer participants for clinical studies was Haffner, who took the perspective of a regulatory agency responsible for reviewing the safety and efficacy of orphan medicines [30, 31]. Haffner argued that orphan medicines should be as well-scrutinized as medicines for more common diseases but recognized that conventional RCTs are not always feasible due to small numbers [30, 31]. Some alternative research methods or design features for demonstrating safety and efficacy that may be acceptable to a regulatory agency were suggested, including the use of multicenter studies, crossover trials, randomized withdrawal trials, open label studies, open protocol studies, and incorporating historical controls or composite or surrogate endpoints [30, 31]. The discussion concerning explanatory evidence generation for clinical interventions for rare diseases continued from these early publications to the present day (Fig. 2). Others elaborated on the issues brought forth by Haffner and offered more suggestions to overcome the challenges related to small numbers and limited feasibility of conventional RCTs, while preserving internal validity and protecting against bias and confounding [2, 4, 18, 32–61].

While participants in our focus groups highlighted the limited feasibility of conventional RCTs because of small sample sizes, there was little emphasis in the focus group discussions on specific strategies that might be used to overcome this challenge. Thus, most of the results presented under the paradigm of explanatory evidence generation are derived from our meta-narrative literature review.

In general, the research methods or study design features that have been proposed in the literature to address small numbers while retaining internal validity and thus an explanatory focus have concentrated on three overarching strategies: (i) enhancing statistical efficiency at the design phase, so that fewer participants are required to conduct a robust evaluation; (ii) using Bayesian rather than frequentist analysis methods, also to reduce the number of participants required; and (iii) making participation more appealing to patients and families by maximizing time spent on the active treatment. Several methodological reviews were published on this topic in the last decade [36, 39, 40, 42, 45, 46, 61], some of which provided more detail about the methods described below; here we focus on the most commonly suggested research designs that focus on minimizing bias to maximize internal validity and explanatory power.

Strategies that have been proposed for enhancing statistical efficiency at the design phase for clinical evaluative studies of rare disease treatments include factorial trials and adaptive designs. Factorial trials are designed to test multiple treatments simultaneously using the same study population, thus reducing the overall number of participants needed [2, 33, 39, 40, 46, 49, 53, 57]. For example, in a 2 × 2 factorial design participants are randomized to either treatment A or control group A, and then randomized again to treatment B or control group B, which effectively reduces the sample size needed to test these two treatments by 50% because the same participants are being randomized [40]. However, authors have pointed out that this reduction in sample size only holds assuming there is no interaction between the treatments being administered concurrently; otherwise, statistical efficiency is lost [40]. Adaptive designs allow flexibility in trial procedures such that changes (“adaptations”) based on interim analyses can be made after trial initiation without undermining the validity of the trial [59]. Two commonly discussed adaptive trial strategies are response-adaptive randomization and group sequential design [36, 40, 46, 53, 59, 61]. Response-adaptive randomization involves modifying treatment assignment probabilities with the accrual of data so that the number of participants randomized to the best-performing treatment arm (“play-the-winner”) is increased and overall sample size is decreased [59]. Group sequential designs do not have a predetermined sample size, rather, small groups of participants are recruited over several phases and data are analyzed at the end of each phase to assess safety, futility, efficacy, or a combination of these until enough data have been accrued to justify study termination [59, 61]. Simulation studies have shown that sequential design approaches may, but do not always, reduce the eventual sample size compared to fixed sample size designs [35, 53, 62]. While adaptive trial strategies are often reported as a means to enhance statistical efficiency, some authors have questioned their usefulness based on the paucity of published practical application in the context of rare diseases [40, 59].

For conventional RCTs with small sample sizes, achieving sufficient statistical power to detect differences in treatment effects, especially when the treatment effect is expected to be modest, is challenging [52]. Several authors have argued (as early as 1995) that Bayesian techniques would be better suited in this context relative to standard frequentist approaches to analysis, because a Bayesian analysis is not as compromised by small numbers and offers more direct conclusions [32, 34, 41, 44, 45, 48, 50–52]. In such approaches, previously collected data or expert opinion is used to generate a prior probability (posterior) distribution for the unknown treatment effect, and Bayes theorem is applied as new data are accumulated to update the posterior distribution for the new treatment and inform clinical practice [48, 52]. As an example, Johnson and colleagues reanalyzed data from an RCT of methotrexate versus placebo in 73 patients with scleroderma, and demonstrated that methotrexate had more favorable odds of being beneficial for patients when a Bayesian approach was applied compared to the non-statistically significant findings obtained through a frequentist approach [32]. While several authors argued that Bayesian statistics offer an alternative approach to the analysis of small numbers of participants, some criticized the subjectivity in establishing prior distributions and were skeptical of the acceptance of results obtained using Bayesian statistics at the regulatory level [34, 36, 45, 48].

It was reported in the literature and in our focus group discussions that there can be a lack of patient/family/clinician acceptance of the possibility of being randomized to a control group, particularly for placebo-controlled studies of treatments for rare diseases where few treatment alternatives exist. Therefore, study designs that make participation more appealing by maximizing time spent on- or guaranteeing provision of- the active treatment have been suggested [4, 33, 36, 38–42, 44–47, 49, 51, 56, 57, 60].

*“I agree with [name]‘s comments that it’s hard to have a placebo-controlled trial. I mean, certainly there has been trials to try to do that. …however, they're very short and really with these, like almost, like even to encounter, to have families agreeable to participate being a placebo for long-term, I think would be very difficult. I think for the short-term, for a few months or a year, families are agreeable, but after that I don’t think they would be agreeable.”* – Physician 2

The randomized placebo-phase design has the same design features of a conventional RCT, except that the time from enrollment in the study to the start of the experimental treatment is randomized for all participants [56]. All participants eventually receive the experimental treatment, and effectiveness is determined based on whether a response is observed sooner among those that received the treatment earlier [56]. Similarly, randomized withdrawal, early escape, and stepped wedge trials reduce time spent in a control arm or ensure that all participants eventually receive the intervention being studied, and have been proposed as alternative approaches to evaluate clinical interventions for rare diseases [40]. Crossover trials and n-of-1 trials also guarantee that participants receive the active treatment, but are different than conventional RCTs in that the treatment sequence is randomized with a washout period in between treatment regimens, such that each participant acts as his or her own control [2, 36, 41, 53, 57]. As some authors reported, n-of-1 trials are often embedded in clinical practice to help healthcare providers determine the best treatments for their patients [2, 36, 57]. While several authors have examined the advantages of crossover and n-of-1 trials, others have discussed the risk of carryover and period effects between phases, and have argued that these designs are generally not suitable for diseases that have an unstable disease course or for interventions that are not fast-acting with reversible effects [2, 18, 33, 36, 39, 44, 46, 53].

The three overarching strategies and associated research methods discussed above are not mutually exclusive, rather there is significant overlap among them in the literature. For example, in addition to being an attractive option for participants, crossover trials are also considered statistically efficient and reduce the number of participants needed because each participant acts as his or her own control [2, 18, 33, 36, 39, 40, 44, 46]. Huang and colleagues have suggested that statistical efficiency could be further enhanced in crossover trials by allowing participants to “escape early” [41]. Similarly, authors have stated that trials using adaptive randomization can be attractive to participants because the likelihood of being randomized to the less effective treatment arm is reduced over time [36, 40, 46, 53, 59, 61]. Bayesian methods are also reported as a common design feature of adaptive trials as a means of improving statistical efficiency [34, 42, 59]. They have also been proposed as a means to combine results from multiple n-of-1 trials and enhance the usability of n-of-1 trial data in answering population-level questions about treatment efficacy and effectiveness [51].

A criticism of explanatory evidence generation reported both in the literature and in focus group discussions was that studies designed to evaluate the efficacy of an intervention typically limit enrolment to a very homogenous group of participants, which strengthens the robustness of the causal interpretation of the findings, but at the expense of a reduction in the external validity or generalizability of study results [4, 18, 44, 60]. Because rare diseases typically exhibit substantial clinical heterogeneity (discussed in the following section), some authors have questioned the suitability of the above-mentioned approaches for evaluating clinical interventions for rare diseases [4, 18, 44, 60]. Additionally, authors have argued that many conventional RCTs and other explanatory studies are short in duration, often due to resource constraints, and do not allow for adequate assessment of long term treatment effects, further compromising external validity [4, 18, 57]. Finally, some authors were concerned that unfamiliar approaches to research design, such as adaptive randomization or n-of-1 trials would not be accepted by regulatory agencies and other policy decision-making bodies [36]. Partly in response to some of these concerns, other research paradigms for evaluating clinical interventions for rare diseases have evolved.

#### Comparative effectiveness/pragmatic evidence generation

It is well established that there is a high degree of clinical heterogeneity among rare disease patients, such that patients with the same specific disease might have drastically different clinical manifestations based on patient characteristics such as age, disease characteristics such as residual enzyme activity levels, or for unknown reasons, and may respond differently to a given intervention [18, 42]. As several authors have discussed, this clinical heterogeneity is often not accounted for in conventional RCTs, and has raised concern among stakeholders about the applicability of study results to patients with clinical manifestations different from those included in RCTs [4, 18, 44, 60].

*“And I find it frustrating in terms of research what I’ve found, and you guys know this, that each case is so unique and different, and so when you read a study or evidence-based research, I find that it, it’s not a guarantee that it’s going to directly correlate to your particular unique situation. So, you have to take that at face value and not think that ‘oh because I read that study and that it is evidence based that this is exactly what’s going to pertain to my situation.”* – Patient/caregiver 4

*“…there’s a huge heterogeneity of this population. There’s people with very severe diseases, people with very mild disease, and this is the nature of enzyme deficiencies. There’s some people that have zero and some people will have, a lot, near normal enzyme activity, so we’re going to get this heterogeneity. And this is one of the big problems, like [name] mentioned, how do we apply this clinically to a larger population of these patients? Are the results, for instance, with infantile-Pompe, how do we relate that to an adult Pompe patient?”* – Physician 5

*“…the way that the trials are designed, very sub, select populations with the actual disease of concern, which is already a narrow disease as it is. It makes it very difficult for us to know where and when these therapies are going to work. And so, when we’re talking about rare diseases, it really has to be linked to not just research, but effectiveness research about natural history and epidemiology. And given the wide degree of heterogeneity with the diseases that we’re dealing with, we’re going into this with a huge degree of uncertainty about whether or not there really is any evidence to support that these therapies are going to work.”* – Policy advisor 2

In response to concerns about the external validity of study results, several authors and focus group participants have advocated for study designs that may compromise internal validity to some extent, by shifting away from the explanatory RCT, in order to address real-world effectiveness [2, 4, 7, 18, 42, 44–47, 55, 57, 58, 63–80].

*“…I think the effort like the Canadian group CIMDRN to look at long-term outcomes, where there’s natural selection of various treatment groups, I think will be very helpful over the long term because of the challenges we have in doing strict study designs, and lack of financial supports for long-term studies. This effect to observational studies and looking at outcome differences in naturally, sort of, selected difference maybe as helpful in rare diseases I think as the designed studies.”* – Physician 1

Almost 10 years after discussions about explanatory evidence generation for clinical interventions for rare diseases emerged in the literature, the research paradigm of comparative effectiveness/pragmatic evidence generation started to develop (first discussion published in 2001). This paradigm was discussed by half (30/60, 50%) of studies included in this review, and was first mentioned by Wilcken in 2001 [7]. Like the previous research paradigm, most of the reports that discussed this paradigm were methodological review articles that focused on rare diseases in general or a group of rare diseases (21/30, 70%), rather than a single rare disease. Wilcken suggested that for some rare diseases, conventional RCTs remained possible, but for others, observational studies with historical controls could be used to evaluate treatment effectiveness [7]. Since that initial publication, many authors have discussed research designs that take a more pragmatic approach to evaluating treatment effectiveness in rare diseases, and often explicitly attempt to include a broader patient population and longer-term observation in natural settings. These designs include: pragmatic clinical trials, observational studies (e.g., cohort studies and registries, case series, case reports), and hybrid designs that incorporate both randomization and systematic observation [2, 4, 18, 42, 44–47, 55, 57, 58, 63–80].

While participants in our focus groups questioned the suitability of explanatory RCTs for establishing effectiveness of clinical interventions for rare diseases, little of the discussion focused on specific solutions to overcome this challenge. Like the previous research paradigm, most of the results presented under the paradigm of comparative effectiveness/pragmatic evidence generation are derived from our meta-narrative literature review.

Incorporating more pragmatic features into RCTs has been suggested as a means to improve external validity while maintaining the element of randomization to help control for unmeasured confounding and maintaining other standard methodological features of explanatory RCTs, such as blinded outcome assessments [18, 45, 57]. These pragmatic RCTs feature design elements that better reflect actual clinical practice, including: enrolling participants with differing clinical presentations, taking into consideration the system of care in which the new treatment will be delivered (e.g., using standard-of-care as a comparator instead of placebo), following participants for a longer period of time, and incorporating outcomes that are meaningful from a patient/care provider standpoint (patient-oriented research will be discussed in the following section) [18, 45, 57]. Authors have criticized pragmatic RCTs because they do still estimate average treatment effects and thus are not necessarily better suited to investigating potential heterogeneity of treatment effects relative to explanatory RCTs [18].

Among the most common observational rare disease research designs discussed in the studies we reviewed are patient registries [4, 18, 42, 47, 58, 64, 65, 67, 72–74, 77, 80] and cohort studies [68, 78]. Because these observational studies do not typically have strict inclusion or exclusion criteria for participants, nor do investigators manipulate participants’ treatment(s), some authors have argued these studies better reflect real-world clinical practice and the clinical heterogeneity that typifies many rare diseases [18, 42, 67, 72]. As reported in the literature, registries have multiple purposes including: evaluating clinical- and/or cost-effectiveness of therapies; monitoring safety of new or existing therapies; evaluating diagnostic tools; monitoring quality of care; and assessing natural history over time [67]. We identified several examples of registries being used to evaluate treatment effectiveness of interventions for rare diseases, for example, enzyme replacement therapy for lysosomal storage disorders [72]. The International Collaborative Gaucher Group Registry was established in 1991 and, at the time of the publication of a paper by Jones and colleagues (2011), had collected longitudinal clinical data for almost 6000 patients [72]. Several authors stated that an additional advantage of registries is that they can be used to identify potential participants for recruitment into future research studies, including clinical trials [18, 67, 73, 76, 77]. Some authors have also suggested that observational patient registries may play an important role in post-market evaluation of interventions for rare diseases by serving as a platform to collect longitudinal clinical and quality of life data [47]. While observational patient registries are an attractive method for the evaluation of longer term outcomes in real-world settings, some authors reported that results remain prone to residual confounding in the absence of randomization, especially confounding by indication (when patient characteristics influencing the choice of treatment also influence the outcome) [18, 44]. A few authors discussed variability in the quality of registry data, as observational patient registries tend to be heterogeneous in the depth of data collection and the definitions applied to included data elements, particularly in the context of the multi-center and sometimes multi-national nature of rare disease research [42, 65]. In addition, some authors described the potentially important influence of complete case ascertainment and data collection on the accuracy of study results, particularly given that registry participation may be associated with receipt of particular treatments or lead to different investigations [67, 73, 81].

In recent years (since 2009), some authors have suggested that elements of both explanatory and observational studies can be combined into “hybrid” study designs that attempt to mitigate challenges faced by both approaches [18, 63, 75]. For example, Vickers and colleagues suggested that the “clinically-integrated randomized trial,” which seeks to integrate randomization into standard clinical care, would be suitable for rare disease research, addressing the threat of confounding while maintaining an element of pragmatism and enhancing generalizability [63]. The key feature of the clinically-integrated randomized trial is that there is no difference between the care a patient routinely receives, follow-up, payment, or documentation (e.g., charting), other than the fact that treatment was assigned randomly with informed consent from participants [63]. In the context of rare diseases, the authors argued that the clinically-integrated randomized trial is attractive because there is often considerable uncertainty about the most effective course of treatment for patients and that trials could easily be conducted worldwide to maximize the number of participants [63]. Another design that incorporates elements of both explanatory and observational approaches and has been suggested in the context of rare diseases is the “cohort multiple randomized controlled trial (cmRCT)” [75]. The cmRCT seeks to enroll an observational cohort of patients, with participants routinely reporting on a minimum set of core outcomes [75, 82]. At the time of enrollment in the cohort, participants give their consent for 1) their longitudinal data to be used in aggregate; and 2) to be randomly selected to participate in potential RCTs of new or existing interventions with the understanding that only those who have been selected to be offered the intervention under study will be contacted [75, 82]. Those who are eligible for the RCT, but who were not randomly selected to be offered the intervention serve as the control group and are not contacted about the study [75, 82]. According to the literature, launching RCTs using this design increases the efficiency of research by accommodating multiple trials and comparison of multiple treatments, allows for longer follow-up of participants, provides pragmatic/real-world evidence, and accommodates clinical heterogeneity by enrolling participants across the clinical spectrum [18, 75, 82]. Concerns that have been raised with these “hybrid” study designs include: potential for confounding and bias in the observational component of the study, and the feasibility of implementing such a study design [18, 75, 82].

Finally, there is discussion in this literature about other observational designs such as case-control studies, small case series and case reports; however, these approaches are not commonly suggested as potential solutions for improving pragmatic evidence generation for establishing effectiveness of treatments for rare diseases. Some authors have suggested that case-control designs, where individuals who have experienced a certain outcome (cases) are matched to and compared with individuals who have not experienced the outcome of interest (controls), are well suited for studying rare diseases, particularly in instances where there could be a long lag time between the treatment and outcome of interest [2, 80]. However, there are concerns about the potential for introducing selection bias in choosing controls [2]. Other authors have argued the importance of case series and case reports in the context of establishing treatment effectiveness for rare diseases [47, 66]. Case series and case reports typically include in-depth information related to clinical manifestations of disease, treatment, and follow-up for a single patient or small group of patients [47, 66]. While authors have acknowledged there are clear limitations in terms of establishing treatment effectiveness, they have argued that this evidence can provide a better understanding of natural history for many rare diseases, and can identify unexpected harms or benefits of treatments, which could be of particular importance for diseases considered “ultra-rare” [47, 66]. Similar to the concept of using case reports as pragmatic evidence, several focus group participants reported relying on some anecdotal evidence to help inform medical-decision making:

*“I think all of the different information is important, and including anecdotal, right? Because we deal with very rare disorders sometimes, and you often go to clinicians who have seen these conditions and have treated them, and may take their point of view about a certain treatment. So, you may say that’s anecdotal, but it may be extremely valuable if there’s only a handful of patients who have received that treatment. So, I think all of the studies and designs, including anecdotal evidence, I personally use that in determining whether I think about a treatment for a patient.”* – Physician 1

*“…sometimes it all depends on the experience of what other people lived. Sometimes people tell you not to go there because they’ve has a bad experience. So, I like to have the bad and the good ones too, and then make my mind and take better decisions.”* – Patient 2

The main criticism in the literature for comparative effectiveness/pragmatic evidence generation is the inherent risk for bias and confounding because of the lack of randomization; however, there have been efforts made to mitigate this risk. As previously discussed, some authors have suggested incorporating pragmatic elements into RCTs [18, 45, 57], while others have proposed methods to overcome challenges in non-randomized studies. For example, Cole and colleagues demonstrated the use of case-control matching using the risk-set method for participants enrolled in the International Collaborative Gaucher Group Registry [69]. The authors applied this method to balance “cases”, i.e., Gaucher patients with skeletal avascular necrosis, and controls according to demographic and clinical factors [69]. Use of propensity scores to match participants has also been suggested as a means of reducing the risk of bias in observational studies of rare diseases [44].

#### Patient-oriented evidence generation

One of the main criticisms, both in the literature and by focus group participants, of highly internally valid, explanatory study designs is their tendency to rely on short-term, and often surrogate, outcomes that are not necessarily clinically meaningful [9].

*“Most of the time study with rare diseases rely on surrogates and the surrogates are selected usually on the basis of biochemical indicators of some biological activity of the treatment. And so, for enzyme replacement therapy, the reduction in the concentration of a substrate in urine or blood is regarded as evidence of a biological pivot, a biological activity, but there’s far too many examples where a surrogate, such as the one I’ve just described, are really, there’s no relationship to what the clinical outcomes are.”* – Policy Advisor 1

*“…I have a concern that sometimes outcome measures are defined by what funding and drug approval [agencies] like FDA want to see, right? [chuckles]. Rather than what the clinician may feel for a particular rare disease is far more important. …it becomes challenging to design appropriate studies and pharma is at the end of the day interested in getting approval and funding approval, and may target outcome measures that are demanded by various bodies rather than perhaps going for the most clinically appropriate outcome measures.”* – Physician 1

Only in the last decade (Fig. 2) has a discussion in the literature emerged regarding the importance of patient-oriented evidence generation in rare diseases (the first appearing in 2010). This discussion emphasizes the need for outcomes that are of direct importance to patients and caregivers. Fifteen of 60 reports (15/60, 25%) discussed issues related to the paradigm of patient-oriented evidence generation, making it the research paradigm with the smallest proportion of literature. The majority of reports that discussed this paradigm were again methodological review articles (13/15, 87%), and the remaining two articles described case examples specific to one rare disease.

Connected to the paradigm of explanatory evidence generation, some authors have suggested the use of surrogate outcomes as proxies for patient-oriented outcomes such as survival or quality of life because they can be measured relatively quickly and require fewer participants to reach statistical efficiency [33, 83–85]. For example, in 2010, Kinder and colleagues reported that functional outcomes such as exercise tolerance, survival, and quality of life were the most salient outcomes to consider for rare lung disease studies because they have undeniable meaning for patients; however, the authors also described the limited feasibility of conducting explanatory RCTs that include these outcomes and argued that surrogate outcomes could therefore be developed and used as proxies for patient-oriented outcomes [33]. Several authors and focus group participants expressed concern about the lack of validation of surrogate outcomes; a clear understanding of the natural history of disease and proposed causal mechanism of a treatment in relation to the disease is needed in order to establish, with reasonable certainty, the relationship between surrogate and patient-oriented outcomes [33, 70, 73, 85, 86].

*“…in order to identify reasonable outcomes measures for any clinical trial, one has to know the what the natural history of the disease is. So, those are major challenges, and what we’re faced with in the pharmaceutical industry, who are anxious to do as short a study as possible, for rare disease almost always use surrogate markers as evidence of effectiveness and the relationship between the surrogate marker and clinical outcome is often completely unknown.”* – Policy Advisor 1

For example, the six-minute walk test (6MWT) is a common surrogate outcome measure used in clinical evaluative studies for many rare diseases [83, 84, 87]. The 6MWT was originally developed for patients with moderate to severe lung disease as a means of assessing overall functional status and as a predictor of morbidity and mortality [88] but has since been used in studies of many rare diseases, including late-onset Pompe disease and Duchenne muscular dystrophy, among others [84, 87]. An important criticism of this extension of its use is the lack of adequate validation to determine if observed changes in the 6MWT reflect meaningful changes for patients [83, 84, 87].

*“For me I think one of the big issues is the outcome measures that we’re trying to document. For instance, with the lysosomal storage diseases, what is the relevance of a 6 minute walk test? What is the clinical relevance of this type of test?”* – Physician 5

Partly in response to concerns about the relevance and validity of surrogate outcomes being used in clinical research for interventions for rare diseases, there has been a shift towards incorporating patient-oriented outcomes in clinical research [4, 42, 45, 74, 89].

*“…because yes, scientific research is important too, but it’s this push-pull dichotomy between the happiness, the living life, just the simple moments, you know, going outside, sitting in the sun, that type of, going down to the beach, those things need to be equally measured…”* – Patient/caregiver 4

*“We need to know more what’s going to happen in terms of lifespan, in terms of morbidity, in terms of the operations these patients are getting, in terms of growth as well. Is this something that we’re seeing improvement?”* – Physician 5

*“I think that [name] made reference to this earlier about the importance of evaluating quality of life. And unfortunately, this is not really done. I don’t know of a single study that has done this rigorously for the diseases that I happen to be involved with or have been. And so, for example, the fact that a child may require an intravenous infusion of some medication that takes six hours of infusion and needs it every week. They’re missing a day of school every week. That’s twenty percent of their schooling! This is never, in my experience, never evaluated. Now that’s not a direct measure of quality of life, but you could easily imagine that it would have a significant indirect impact on quality of life.”* – Policy Advisor 1

In the literature and among our focus group participants, much of the discussion regarding patient-oriented outcomes has focused on developing outcomes that are meaningful based on the lived experiences of patients and their caregivers [18, 42, 74, 89]. Tudur Smith and colleagues used the example of juvenile idiopathic arthritis to demonstrate that clinical research initially focused on outcomes related to clinical disease activity and disease damage, but more recently has shifted to identifying and validating outcomes that are most important to patients and parents, such as health related quality of life, functional assessments, and pain assessments [45]. Basch and Bennett advocated for the use of patient-reported outcomes in clinical studies for interventions for rare disease as the best measurement tools for how a patient feels and functions [89]. Participants in our focus groups also expressed a desire for researchers to incorporate outcomes beyond those directly related to the patient, including parent- and family-related outcomes.

“One quick comment about the whole family because I know, obviously, a lot of this is directed towards the patient, the person with [disease], but it’s, you know, so linked and so connected, that I find there’s a direct, you know, effect on the child through the parents, so I’d like to see more supports, research for the parents that are also kind of surviving through this…” – Patient/caregiver 4

A common criticism is that many outcome measures, including patient-oriented outcome measures, have not been validated or standardized for the population of interest, leading to questions about the applicability of study results [4, 42, 70].

*“… we know that some of these tests or some of the questionnaires have not been standardized for these particular populations, and we’re faced with always the question is it clinically relevant for these patients? I think overall, there’s agreement that they are, but we run into this problem all the time with, you know, Pompe or the different MPS’ because there hasn’t been long enough natural history studies, there has not been standardization of these tests, so we’re choosing these measuring tools for these particular studies without really knowing if they’re the best tools. And this is very relevant for the quality of life questionnaires, we sometimes use the SF36 or we use specific pain criteria, APPT or something like that, but we haven’t actually standardized this for these populations, so we don’t actually know if what we’re measuring is clinically relevant.”* – Physician 5

In response to this criticism, some researchers have begun to identify/develop and validate standard sets of outcome measures that can be used in clinical research evaluating treatment effectiveness in their populations [4, 45, 76]. Another concern that has been raised with respect to outcomes is that it may not be possible to use the same outcome measure within the same disease if there is substantial clinical heterogeneity among patients [4, 42, 45, 84, 89]. Some authors and focus group participants also noted that clinical heterogeneity has implications for identifying the minimal clinically important difference [42].

*“…the main trial showed an improvement of 22.5 meters after 6 months in the six-minute walk test, which there’s quite a variability in outcomes depending on which patients you’re looking at, but that the average improvement. What does that really mean is a very difficult decision because for somebody that is walking perhaps 300 meters in six minutes and improves by 22.5 meters, that’s probably not clinically significant, if we’re just looking at a six-minute walk test. But, if someone is not very mobile at all and has that improvement, we might actually have a more clinically significant impact with that treatment.”* – Physician 5

Finally, some focus group participants expressed concern about balancing subjective outcomes (e.g., patient-reported quality of life) with more objective outcomes (e.g., biomarkers of disease progression) because of possible placebo effects with patient-reported outcomes.

*“I think there needs to be a combination of objective and subjective outcome measures and quality of life measures because, certainly, quality of life is extremely important, but my sense is that it’s a lot more vulnerable to placebo effect. As well, just in the sense that a lot of these families are extremely invested in being on their therapy because it is their only therapeutic option. And so by relying on quality of life measures very heavily, I think we can end up advocating for treatment for patients that aren’t really clinically benefitting.”* – Physician 6

## Discussion

Randomized controlled trials have long been considered the ‘gold standard’ in evidence-based medicine due to their superior ability to maximize internal validity [5]. However, our review and focus group findings describe criticisms of conventional explanatory RCTs to establish treatment effectiveness for rare disease therapies. There was agreement across the focus group interviews and with the literature we reviewed that the main challenges in generating robust treatment efficacy and effectiveness evidence for rare diseases includes: i) limitations in recruiting a sufficient sample size to achieve planned statistical power for many rare diseases, especially those with a low prevalence such as MPS; ii) difficulties in accounting for characteristic clinical heterogeneity of many rare diseases; and iii) frequent reliance on short-term, surrogate outcomes whose clinical relevance is often unclear. We mapped these three perceived challenges and associated methodological solutions to three interrelated research paradigms that emerged from our data: i) explanatory evidence generation, ii) comparative effectiveness/pragmatic evidence generation, and iii) patient-oriented evidence generation. Discussions related to explanatory evidence generation were the first to arise in the rare disease literature (in 1992) and have persisted through 2016, with 58% (35/60) of the reports we reviewed examining this research paradigm. The paradigm of comparative effectiveness/pragmatic evidence generation, which was discussed in 50% (30/60) of reports, emerged in the literature in the early 2000s and has also persisted through 2016, with a substantial increase in the number of reports in the literature over the last decade. The paradigm of patient-oriented evidence generation developed more recently in the literature (beginning in 2010) and has been discussed in 25% (15/60) of reports included in this review. Based on the year of publication for the included studies, there appears to be a shift in perspectives over time with increased criticism of conventional explanatory RCTs and associated recognition of the importance of pragmatic and patient-oriented evidence generation in the context of establishing treatment effectiveness for rare diseases.

Several methodological solutions have been suggested within each research paradigm to address the perceived challenges that were identified both in the literature and by our focus group participants. For explanatory evidence generation, the potential solutions include: study designs that incorporate elements to improve statistical efficiency and reduce the required sample size (e.g., factorial trials, adaptive designs, applying Bayesian statistical methods), and study designs that ensure receipt of or maximize time spent on active treatment to help boost participation (e.g., randomized placebo-phase designs, crossover/N-of-1 trials). For comparative effectiveness/pragmatic evidence generation, study designs or features that have been proposed to improve the external validity of study results include: incorporating pragmatic elements into conventional RCTs, registries/cohort studies, and hybrid designs such as cmRCTs. For patient-oriented evidence generation, authors and focus group participants suggested that incorporating outcomes that are considered important by patients and their caregivers (e.g., health-related quality of life) is critical to improve the applicability of study results.

Notably, though numerous non-conventional study designs were described in the literature we reviewed, few of the suggested approaches appear to have been applied successfully in the context of rare diseases. Only 28% (17/60) reports included in this review were considered applications or case examples of a specific research method. As suggested by Gupta and colleagues, the paucity of real-world application of these designs, particularly the non-conventional explanatory RCT designs, may be related to a lack of acceptance of unfamiliar study designs [36]. New therapies for many rare diseases are rapidly developing, so there is an increasing opportunity to apply some of these non-conventional study design strategies to evaluate efficacy and effectiveness of emerging treatments for rare diseases [Stockler-Ipsiroglu et al. Innovations in therapies and evidence creation for inborn errors of metabolism, in progress].

Among the suggested methodological strategies, there are tradeoffs with respect to internal and external validity, some of which may be exacerbated in the context of rare diseases. For example, external validity is compromised in many explanatory RCTs in favour of maintaining strong internal validity to reduce potential bias and confounding. In addition, because of the small number of individuals available to participate in research, relying on randomization procedures to balance patient characteristics (both known and unknown) will not always be successful. By contrast, study designs that can better accommodate clinical heterogeneity and enhance external validity may introduce a risk of confounding and bias. And while external validity can be compromised if the outcomes(s) included in a study are not considered important by clinicians and patients, many patient-oriented outcome measures require additional validation and long-term follow-up. With these tradeoffs in mind, strategies for both comparative effectiveness/pragmatic and patient-oriented evidence generation are increasingly being recognized as important for investigating the effectiveness of treatments for rare diseases, with explanatory RCTs becoming less dominant in the literature in recent years.

The results of our meta-narrative review corroborate the conclusions of methodological reviews that have focused on approaches to generating evidence for interventions for rare diseases [36, 39, 40, 42, 45, 46]. To our knowledge, our study is the first to incorporate stakeholder perspectives in addition to data from the published literature and to include a description of how perspectives have evolved over time using a meta-narrative review. Many of the approaches described in previously published reviews are specific to explanatory evidence generation. For example, both Gupta and colleagues and Cornu and colleagues provide algorithms that could be used by researchers to facilitate decision-making about which explanatory trial design to apply for a particular rare disease research question [36, 40]. Previous reviews included limited discussion of pragmatic evidence generation, with the exception of observational methods such as registries or cohort studies [42, 46]. Gagne and colleagues were the only authors among our reviewed studies to include an in-depth discussion about strategies that could be used to mitigate bias and confounding in observational studies of interventions for rare diseases [46]. Previously published reviews rarely mentioned patient-oriented outcomes in the context of evidence generation related to rare diseases.

Our work is not without limitations. The search strategy that was developed for the meta-narrative portion of this study was not exhaustive, so there is a possibility that some literature may have been missed. However, our intention was to identify key literature on this topic. In addition, we only had a single reviewer (KT) who determined study eligibility, which could have led to selection bias in the articles chosen; however, clear inclusion and exclusion criteria were used and the study team met several times to review selected literature and discuss emerging findings. We only conducted three focus group interviews with a relatively small, convenience sample of participants; consequently, we may have missed some perspectives. Our patient/caregiver focus group was particularly narrow in its focus on a single group of rare diseases. Because we were able to leverage an existing meeting of an otherwise geographically dispersed group of patients and families with MPS, an advantage of our approach was the ability to conduct an in-person focus group interview and thus ascertain the views of the participants more fully. However, some of the perspectives may have been specific to that disease group and future research could explore the perspectives of patients and families with other rare diseases, including those with a relatively higher prevalence for whom conventional explanatory studies might be more feasible (e.g., cystic fibrosis).

## Conclusions and future directions

Through our meta-narrative literature review and focus group interviews we identified several perceived challenges and potential solutions for generating robust treatment effectiveness evidence for rare diseases according to three interrelated research paradigms: explanatory, comparative effectiveness/pragmatic, and patient-oriented evidence generation. Over time, there has been more recognition that observational studies, such as patient registries and cohort studies, are important approaches for clinical evaluative research in the context of rare diseases to address gaps in comparative effectiveness/pragmatic and patient-oriented evidence generation. Developing better methods to mitigate potential bias and confounding would increase the value of these approaches for establishing treatment effectiveness in the rare disease context. From a policy perspective, there is a need for inclusive discussions amongst patients and their families, clinicians, and policy advisors, including those involved in regulatory and reimbursement decision-making about interventions for rare diseases, in order to identify solutions that meet the needs of all stakeholder groups. Finally, little research has been done with respect to developing knowledge synthesis methods that consider the challenges faced in generating robust evidence for rare diseases. Future directions for our work include developing a framework to expand current evidence synthesis practices to take into consideration many of the concepts discussed in this paper.

## Additional files

Additional file 1: Search strategies for electronic databases. (DOCX 64 kb)

Additional file 2: List of articles included in meta-narrative literature review. (XLSX 53 kb)

## Abbreviations

6MWT: Six-minute walk test
cmRCT: Cohort multiple randomized controlled trial
RCT: Randomized controlled trial
