# Supplementary material for: Using a meta-narrative literature review and focus groups with key stakeholders to identify perceived challenges and solutions for generating robust evidence on the effectiveness of treatments for rare diseases
Source: Orphanet J Rare Dis. 2018 Jun 28;13:104. doi: 10.1186/s13023-018-0851-1 (PMC6022712; doi:10.1186/s13023-018-0851-1)
Supplement: Supplementary file 1 — Search strategies for electronic databases. (DOCX 64 kb) [file 13023_2018_851_MOESM1_ESM.docx]

**Additional File 1 – Search strategies**

MEDLINE (Ovid MEDLINE(R) In-Process & Other Non-Indexed Citations and Ovid MEDLINE(R) 1946 to June 21, 2017)

1. exp Epidemiologic Study Characteristics as Topic/
2. Research Design/
3. Patient Outcome Assessment/
4. Treatment Outcome/
5. Rare Diseases/
6. Orphan Drug Production/
7. 1 or 2 or 3 or 4
8. 5 or 6
9. 7 and 8
10. limit 9 to English language

EMBASE (Embase Classic + Embase 1947 to June 21, 2017)

1. *epidemiology/
2. *controlled study/
3. *clinical trial/
4. *randomized controlled trial/
5. *observational study/
6. *methodology/
7. *outcome assessment/
8. *treatment outcome/
9. rare disease/
10. orphan drug/
11. rare disease*.tw.
12. orphan disease*.tw.
13. 1 or 2 or 3 or 4 or 5 or 6 or 7 or 8
14. 9 or 10 or 11 or 12
15. 13 and 14

Pubmed

1. (((epidemiologic study characteristics as topic[MeSH Terms]) OR research design[MeSH Terms]) OR patient outcomes assessment[MeSH Terms]) OR treatment outcome[MeSH Terms]
2. (rare diseases[MeSH Terms]) OR orphan drug production[MeSH Terms]
3. 1 and 2
